# Nonlinear Dendritic Coincidence Detection for Supervised Learning

**DOI:** 10.3389/fncom.2021.718020

**Published:** 2021-08-04

**Authors:** Fabian Schubert, Claudius Gros

**Affiliations:** Institute for Theoretical Physics, Goethe University Frankfurt am Main, Frankfurt am Main, Germany

**Keywords:** dendrites, pyramidal neuron, plasticity, coincidence detection, supervised learning

## Abstract

Cortical pyramidal neurons have a complex dendritic anatomy, whose function is an active research field. In particular, the segregation between its soma and the apical dendritic tree is believed to play an active role in processing feed-forward sensory information and top-down or feedback signals. In this work, we use a simple two-compartment model accounting for the nonlinear interactions between basal and apical input streams and show that standard unsupervised Hebbian learning rules in the basal compartment allow the neuron to align the feed-forward basal input with the top-down target signal received by the apical compartment. We show that this learning process, termed coincidence detection, is robust against strong distractions in the basal input space and demonstrate its effectiveness in a linear classification task.

## 1. Introduction

In recent years, a growing body of research has addressed the functional implications of the distinct physiology and anatomy of cortical pyramidal neurons (Spruston, 2008; Hay et al., 2011; Ramaswamy and Markram, 2015). In particular, on the theoretical side, we saw a paradigm shift from treating neurons as point-like electrical structures toward embracing the entire dendritic structure (Larkum et al., 2009; Poirazi, 2009; Shai et al., 2015). This was mostly due to the fact that experimental work uncovered dynamical properties of pyramidal neuronal cells that simply could not be accounted for by point models (Spruston et al., 1995; Häusser et al., 2000).

An important finding is that the apical dendritic tree of cortical pyramidal neurons can act as a separate nonlinear synaptic integration zone (Spruston, 2008; Branco and Häusser, 2011). Under certain conditions, a dendritic *Ca*^2+^ spike can be elicited that propagates toward the soma, causing rapid, bursting spiking activity. One of the cases in which dendritic spiking can occur was termed ‘backpropagation-activated *Ca*^2+^ spike firing' (“BAC firing”): A single somatic spike can backpropagate toward the apical spike initiation zone, in turn significantly facilitating the initiation of a dendritic spike (Stuart and Häusser, 2001; Spruston, 2008; Larkum, 2013). This reciprocal coupling is believed to act as a form of coincidence detection: If apical and basal synaptic input co-occurs, the neuron can respond with a rapid burst of spiking activity. The firing rate of these temporal bursts exceeds the firing rate that is maximally achievable under basal synaptic input alone, therefore representing a form of temporal coincidence detection between apical and basal input.

Naturally, these mechanisms also affect plasticity, and thus learning within the cortex (Sjöström and Häusser, 2006; Ebner et al., 2019). While the interplay between basal and apical stimulation and its effect on synaptic efficacies is subject to ongoing research, there is evidence that BAC-firing tends to shift plasticity toward long-term potentiation (LTP) (Letzkus et al., 2006). Thus, coincidence between basal and apical input appears to also gate synaptic plasticity.

In a supervised learning scheme, where the top-down input arriving at the apical compartment acts as the teaching signal, the most straight-forward learning rule for the basal synaptic weights would be derived from an appropriate loss function, such as a mean square error, based on the difference between basal and apical input, i.e., *I*_*p*_ − *I*_*d*_, where indices *p* and *d* denote ‘proximal' and ‘distal', in equivalence to basal and apical. Theoretical studies have investigated possible learning mechanisms that could utilize an intracellular error signal (Urbanczik and Senn, 2014; Schiess et al., 2016; Guerguiev et al., 2017). However, a clear experimental evidence for a physical quantity encoding such an error is—to our knowledge—yet to be found. On the other hand, Hebbian-type plasticity is extensively documented in experiments (Gustafsson et al., 1987; Debanne et al., 1994; Markram et al., 1997; Bi and Poo, 1998). Therefore, our work is based on the question of whether the nonlinear interactions between basal and apical synaptic input could, when combined with a Hebbian plasticity rule, allow a neuron to learn to reproduce an apical teaching signal in its proximal input.

We investigate coincidence learning by combining a phenomenological model that generates the output firing rate as a function of two streams of synaptic input (subsuming basal and apical inputs) with classical Hebbian, as well as BCM-like plasticity rules on basal synapses. In particular, we hypothesized that this combination of neural activation and plasticity rules would lead to an increased correlation between basal and apical inputs. Furthermore, the temporal alignment observed in our study could potentially facilitate apical inputs to act as top-down teaching signals, without the need for an explicit error-driven learning rule. Thus, we also test our model in a simple linear supervised classification task and compare it with the performance of a simple point neuron equipped with similar plasticity rules.

## 2. Model

### 2.1. Compartamental Neuron

The neuron model used throughout this study is a discrete-time rate encoding model that contains two separate input variables, representing the total synaptic input current injected arriving at the basal (proximal) and apical (distal) dendritic structure of a pyramidal neuron, respectively. The model is a slightly simplified version of a phenomenological model proposed by Shai et al. (2015). Denoting the input currents *I*_*p*_ (proximal) and *I*_*d*_ (distal), the model is written as

(1) $$\begin{array}{c} y\left(t\right)=\alpha \sigma \left(I_{p}\left(t\right)-\theta_{p0}\right)\left[1-\sigma \left(I_{d}\left(t\right)-\theta_{d}\right)\right]\\ +\sigma \left(I_{d}\left(t\right)-\theta_{d}\right)\sigma \left(I_{p}\left(t\right)-\theta_{p1}\right) \end{array}$$

(2) $$\begin{array}{ll} \sigma \left(x\right) & \equiv \frac{1}{1+\exp\left(-4x\right)}. \end{array}$$

Here, θ_*p*0_ > θ_*p*1_ and θ_*d*_ are threshold variables with respect to proximal and distal inputs. Equation (1) defines the firing rate *y* as a function of *I*_*p*_ and *I*_*d*_. Note that the firing rate is normalized to take values within *y* ∈ [0, 1]. In the publication by Shai et al. (2015), firing rates varied between 0 and 150Hz. High firing rates typically appear in the form of bursts of action potentials, lasting on the order of 50–100ms (Larkum et al., 1999; Shai et al., 2015). Therefore, since our model represents “instantaneous” firing rate responses to a discrete set of static input patterns, we conservatively estimate the time scale of our model to be on the order of tenths of seconds.

In general, the input currents *I*_*p*_ and *I*_*d*_ are meant to comprise both excitatory and potential inhibitory currents. Therefore, we did not restrict the sign of of *I*_*p*_ and *I*_*d*_ to positive values. Moreover, since we chose the thresholds θ_*p*0_ and θ_*d*_ to be zero, *I*_*p*_ and *I*_*d*_ should be rather seen as a total external input relative to intrinsic firing thresholds.

Note that the original form of this phenomenological model by Shai et al. (2015) is of the form

(3) $$\begin{array}{l} y\left(I_{p},I_{d}\right)=\sigma \left(I_{p}-A\sigma \left(I_{d}\right)\right)\left[1+B\sigma \left(I_{d}\right)\right], \end{array}$$

where σ denotes the same sigmoidal activation function. This equation illustrates that *I*_*d*_ has two effects: It shifts the basal activation threshold by a certain amount (here controlled by the parameter *A*) and also multiplicatively increases the maximal firing rate (to an extent controlled by *B*). Our equation mimics these effects by means of the two thresholds θ_*p*0_ and θ_*p*1_, as well as the value of α relative to the maximal value of y (which is 1 in our case).

**Figure 1:**
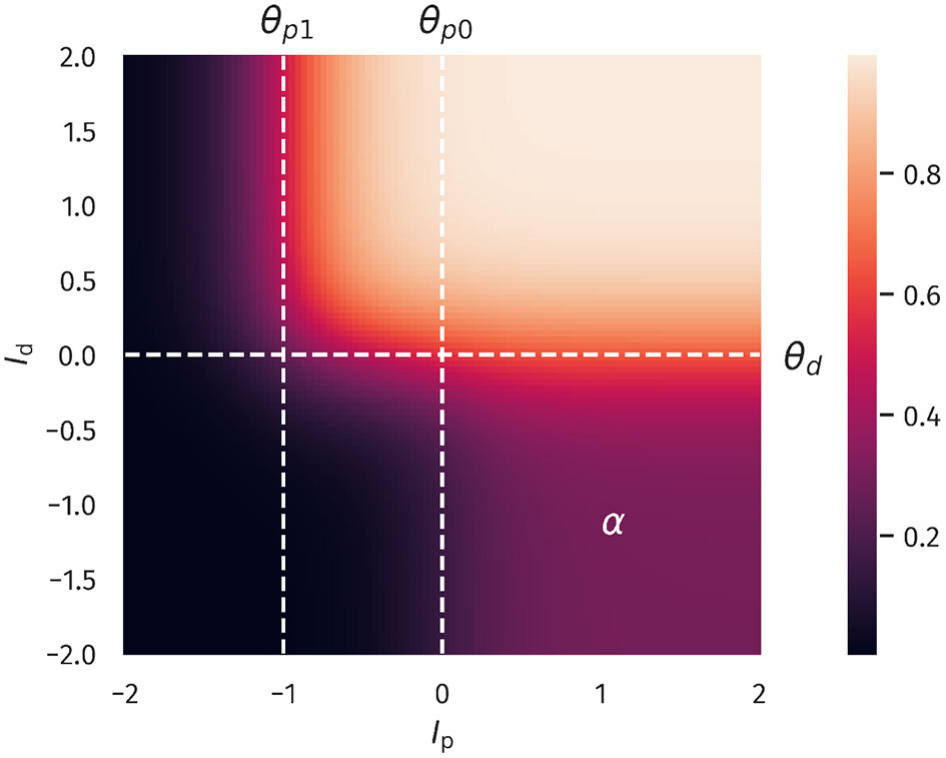
Two-compartment rate model. The firing rate as a function of proximal and distal inputs *I*_*p*_ and *I*_*d*_, see Equation (1). The thresholds θ_*p*0_, θ_*p*1_ and θ_*d*_ define two regions of neural activity, with a maximal firing rate of 1 and a plateau in the lower-left quadrant with a value of α = 0.3. That is, the latter region can achieve 30% of the maximal firing rate.

Overall, Equation (1) describes two distinct regions of neural activation in the (*I*_*p*_, *I*_*d*_)-space which differ in their maximal firing rates, which are set to 1 and α, where 0 < α <1. A plot of Equation (1) is shown in Figure 1.

When both input currents *I*_*d*_ and *I*_*p*_ are large, that is, larger than the thresholds θ_*d*_ and θ_*p*1_, the second term in Equation (1) dominates, which leads to *y* ≈ 1. An intermediate activity plateau, of strength α emerges in addition when *I*_*p*_ > θ_*p*0_ and *I*_*d*_ < θ_*d*_. As such, the compartment model Equation (1) is able to distinguish neurons with a normal activity level, here encoded by α = 0.3, and strongly bursting neurons, where the maximal firing rate is unity. The intermediate plateau allows neurons to process the proximal inputs *I*_*p*_ even in the absence of distal stimulation. The distal current *I*_*d*_ acts therefore as an additional modulator.

In our numerical experiments, we compare the compartment model with a classical point neuron, as given by

(4) $$\begin{array}{l} y\left(t\right)=\sigma \left(I_{p}\left(t\right)+I_{d}\left(t\right)-\theta \right). \end{array}$$

The apical input *I*_*d*_ is generated ‘as is', meaning it is not dynamically calculated as a superposition of multiple presynaptic inputs. For concreteness, we used

(5) $$\begin{array}{l} I_{d}\left(t\right)=n_{d}\left(t\right)x_{d}\left(t\right)-b_{d}\left(t\right), \end{array}$$

where *n*_*d*_(*t*) is a scaling factor, *x*_*d*_(*t*) a discrete time sequence, which represents the target signal to be predicted by the proximal input, and *b*_*d*_(*t*) a bias. In our experiments, we chose *x*_*d*_ according to the prediction task at hand, see Equations (17), (19), and (20).

Note that *n*_*d*_ and *b*_*d*_ are time dependent since they are subject to adaptation processes, which will be described in the next section. Similarly, the proximal input *I*_*p*_(*t*) is given by

(6) $$\begin{array}{l} I_{p}\left(t\right)=n_{p}\left(t\right)\overset{N}{\underset{i=1}{\sum }}x_{p,i}\left(t\right)w_{i}\left(t\right)-b_{p}\left(t\right), \end{array}$$

where *N* is the number of presynaptic afferents, *x*_*p,i*_(*t*) the corresponding sequences, *w*_*i*_(*t*) the synaptic efficacies and *n*_*p*_(*t*) and *b*_*p*_(*t*) the (time dependent) scaling and bias. Tyical values for the parameters used throughout this study are presented in Table 1.

**Table 1:** Model parameters, as defined in sections 2.1, 2.3.

θ_*p*0_	0	$V_{d}^{t}$	0.25
θ_*p*1_	−1	μ_*b*_	10^−3^
θ_*d*_	0	μ_*n*_	10^−4^
α	0.3	μ_av_	5·10^−3^
μ_*w*_	5·10^−5^	$I_{p}^{t}$	0
ϵ	0.1	$I_{d}^{t}$	0
$V_{p}^{t}$	0.25		

### 2.2. Homeostatic Parameter Regulation

The bias variables entering the definitions (Equations 5, 6) of the distal proximal current, *I*_*d*_ and *I*_*p*_, are assumed to adapt according to

(7) $$\begin{array}{ll} b_{p}\left(t+1\right) & =b_{p}\left(t\right)+\mu_{b}\left[I_{p}\left(t\right)-I_{p}^{t}\right] \end{array}$$

(8) $$\begin{array}{ll} b_{d}\left(t+1\right) & =b_{d}\left(t\right)+\mu_{b}\left[I_{d}\left(t\right)-I_{d}^{t}\right], \end{array}$$

where $I_{p}^{t}=0$ and, $I_{d}^{t}=0$ are preset targets and $1/\mu_{b}=10^{3}$ is the timescale for the adaption. Since this is a slow process, over time, both the distal and the proximal currents, *I*_*d*_ and *I*_*p*_, will approach a temporal mean equal to $I_{p}^{t}$ and $I_{d}^{t}$, respectively, while still allowing the input to fluctuate. The reason for choosing the targets to be zero lies in the fact that we expect a neuron to operate in a dynamical regime that can reliably encode information from its inputs. In the case of our model, this implies that neural input should be distributed close to the threshold (which was set to zero in our case), such that fluctuations in the can have an effect on the resulting neural activity. See e.g., Bell and Sejnowski (1995) and Triesch (2007) for theoretical approaches to optimizing gains and biases based on input and output statistics. Hence, while we chose the mean targets of the input to be the same as the thresholds, this is not a strict condition, as relevant information in the input could also be present in parts of the input statistics that significantly differ from its actual mean (for example in the case of a heavily skewed distribution).

Adaptation rules for the bias entering a transfer function, such as Equations (8), (7), have the task to regulate overall activity levels. The overall magnitude of the synaptic weights, which are determined by synaptic rescaling factors, here *n*_*d*_ and *n*_*p*_, as defined in Equations (5), (6), will regulate in contrast the variance of the neural activity, and not the average level (Schubert and Gros, 2021). In this spirit we consider

(9) $$\begin{array}{ll} n_{d}\left(t+1\right) & =n_{d}\left(t\right)+\mu_{n}\left[V_{d}^{t}-{\left(I_{d}\left(t\right)-{Ĩ}_{d}\left(t\right)\right)}^{2}\right] \end{array}$$

(10) $$\begin{array}{ll} n_{p}\left(t+1\right) & =n_{p}\left(t\right)+\mu_{n}\left[V_{p}^{t}-{\left(I_{p}\left(t\right)-{Ĩ}_{p}\left(t\right)\right)}^{2}\right] \end{array}$$

(11) $$\begin{array}{ll} {Ĩ}_{d}\left(t+1\right) & =\left(1-\mu_{\text{av}}\right){Ĩ}_{d}\left(t\right)+\mu_{\text{av}}I_{d}\left(t\right) \end{array}$$

(12) $$\begin{array}{ll} {Ĩ}_{p}\left(t+1\right) & =\left(1-\mu_{\text{av}}\right){Ĩ}_{p}\left(t\right)+\mu_{\text{av}}I_{p}\left(t\right). \end{array}$$

Here, $V_{p}^{t}$ and $V_{p}^{t}$ define targets for the temporally averaged variances of *I*_*p*_ and *I*_*d*_. The dynamic variables Ĩ_*p*_ and Ĩ_*d*_ are simply low-pass filtered running averages of *I*_*p*_ and *I*_*d*_. Overall, the framework specified here allows the neuron to be fully flexible, as long as the activity level and its variance fluctuate around preset target values (Schubert and Gros, 2021).

Mapping the control of the mean input current to the biases and the control of variance to the gains is, in a sense, an idealized case of the more general notion of dual homeostasis. As shown by Cannon and Miller (2017), the conditions for a successful control of mean and variance by means of gains and biases are relatively loose: Under certain stability conditions, a combination of two nonlinear functions of the variable that is to be controlled can yield a dynamic fixed point associated with a certain mean and variance. In fact, a possible variant of dual homeostasis could potentially be achieved by coupling the input gains to a certain firing rate (which is a non-linear function of the input), while biases are still adjusted to a certain mean input. This, of course, would make it harder to predict the variance of the input resulting from such an adaptation, since it would not enter the equations as a simple parameter that can be chosen a priori (as it is the case for Equations 11, 12).

A list of the parameter values used throughout this investigation is also given in Table 1. Our choices of target means and variances are based on the assumption that neural input should be tuned toward a certain working regime of the neural transfer function. In the case of the presented model, this means that both proximal and distal input cover an area where the nonlinearities of the transfer function are reflected without oversaturation.

### 2.3. Synaptic Plasticity

The standard Hebbian plasticity rule for the proximal synaptic weights is given by

(13) $$\begin{array}{ll} w_{i}\left(t+1\right) & =w_{i}\left(t\right)+\mu_{w}\left[(x_{p,i}\left(t\right)-{\tilde{x}}_{p,i}\left(t\right)\right)\left(y\left(t\right)-ỹ\right)-\epsilon w_{i}\left(t)\right] \end{array}$$

(14) $$\begin{array}{ll} {\tilde{x}}_{p,i}\left(t+1\right) & =\left(1-\mu_{\text{av}}\right){\tilde{x}}_{p,i}\left(t\right)+\mu_{\text{av}}x_{p,i}\left(t\right) \end{array}$$

(15) $$\begin{array}{ll} ỹ\left(t+1\right) & =\left(1-\mu_{\text{av}}\right)ỹ\left(t\right)+\mu_{\text{av}}y\left(t\right) \end{array}$$

The trailing time averages ${\tilde{x}}_{p,i}$ and ỹ, respectively of the presynaptic basal activities, *x*_*p,i*_, and of the neural firing rate *y*, enter the Hebbian learning rule (13) as reference levels. Pre- and post-synaptic neurons are considered to be active/inactive when being above/below the respective trailing averages. This is a realization of the Hebbian rule proposed by Linsker (1986). The timescale of the averaging, 1/μ_av_, is 200 time steps, see Table 1. As discussed in section 2.1, a time step can be considered to be on the order of 100ms, which equates to an averaging time of about 20s. Generally, this is much faster than the timescales on which metaplasticity, i.e., adaptation processes affecting the dynamics of synaptic plasticity itself, are believed to take place, which are on the order of days (Yger and Gilson, 2015). However, it should be noted that our choice of the timescale of the averaging process used in our plasticity model is motivated mostly by considerations regarding the overall simulation time: Given enough update steps, the same results could be achieved by an arbitrarily slow averaging process.

Since classical Hebbian learning does not keep weights bounded, we use an additional proportional decay term ϵ*w*_*i*_ which prevents runaway growth using ϵ = 0.1. With $1/\mu_{w}=2\cdot 10^{4}$, learning is assumed to be considerably slower, as usual for statistical update rules. For comparative reasons, the point neuron model (Equation 4) is equipped with the same plasticity rule for the proximal weights as Equation (13).

Apart from classical Hebbian learning, we also considered a BCM-like learning rule for the basal weights (Bienenstock et al., 1982; Intrator and Cooper, 1992). The form of the BCM-rule used here reads

(16) $$\begin{array}{l} w_{i}\left(t+1\right)=w_{i}\left(t\right)+\mu_{w}[y\left(y-\theta_{M}\right)x_{i}-\epsilon w_{i}], \end{array}$$

where θ_*M*_ is a threshold defining a transition from long-term potentiation (LTP) to long-term depression (LTD) and, again, ϵ is a decay term on the weights preventing unbounded growth. In the variant introduced by Law and Cooper (1994), the sliding threshold is simply the temporal average of the squared neural activity, $\theta_{M}=\langle y^{2}\rangle$. In practice, this would be calculated as a running average, thereby preventing the weights from growing indefinitely.

However, for our compartment model, we chose to explicitly set the threshold to be the mean value between the high- and low-activity regime in our compartment model, i.e., θ_*M*_ = (1 + α)/2. By doing so, LTP is preferably induced if both basal and apical input is present at the same time. Obviously, for the point model, the reasoning behind our choice of θ_*M*_ did not apply. Still, to provide some level of comparability, we also ran simulations with a point model where the sliding threshold was calculated as a running average of *y*^2^.

## 3. Results

### 3.1. Unsupervised Alignment Between Basal and Apical Inputs

As a first test, we quantify the neuron's ability to align its basal input to the apical teaching signal. This can be done using the Pearson correlation coefficient ρ[*I*_*p*_, *I*_*d*_] between the basal and apical input currents. We determined ρ[*I*_*p*_, *I*_*d*_] after the simulation, which involves all plasticity mechanisms, both for the synaptic weights and the intrinsic parameters. The input sequences *x*_*p,i*_(*t*) is randomly drawn from a uniform distribution, in [0, 1], which is done independently for each *i* ∈ [1, *N*].

For the distal current *I*_*d*_(*t*) to be fully ‘reconstructable' by the basal input, *x*_*d*_(*t*) has to be a linear combination

(17) $$\begin{array}{ll} x_{d}\left(t\right) & =\overset{N}{\underset{i=1}{\sum }}a_{i}x_{p,i}\left(t\right) \end{array}$$

of the *x*_*p,i*_(*t*), where the *a*_*i*_ are the components of a random vector *a* of unit length.

Given that we use with Equation (13) a Hebbian learning scheme, one can expect that the direction and the magnitude of the principal components of the basal input may affect the outcome of the simulation significantly: A large variance in the basal input orthogonal to the ‘reconstruction vector' **a** is a distraction for the plasticity. The observed temporal alignment between *I*_*p*_ and *I*_*d*_ should hence suffer when such a distraction is present.

In order to test the effects of distracting directions, we applied a transformation to the input sequences *x*_*p,i*_(*t*). For the transformation, two parameters are used, a scaling factor *s* and the dimension *N*_dist_ of the distracting subspace within the basal input space. The *N*_dist_ randomly generated basis vectors are orthogonal to the superposition vector **a**, as defined by Equation (17), and to each others. Within this *N*_dist_-dimensional subspace, the input sequences *x*_*p,i*_(*t*) are rescaled subsequently by the factor *s*. After the learning phase, a second set of input sequences *x*_*p,i*_(*t*) and *x*_*d*_(*t*) is generated for testing purposes, using the identical protocol, and the cross correlation ρ[*I*_*p*_, *I*_*d*_] evaluated. During the testing phase plasticity is turned off.

The overall aim of our protocol is to evaluate the degree ρ[*I*_*p*_, *I*_*d*_] to which the proximal current *I*_*p*_ aligns in the temporal domain to the distal input *I*_*d*_. We recall that this is a highly non-trivial question, given that the proximal synaptic weights are adapted via Hebbian plasticity, see Equation (13). The error ${\left(I_{p}-I_{d}\right)}^{2}$ does not enter the adaption rules employed. Results are presented in **Figure 3** as a function of the distraction parameters *s* and *N*_dist_ ∈ [0, *N* − 1]. The total number of basal inputs is *N* = 100.

For comparison, in **Figure 3** data for both the compartment model and for a point neuron are presented (as defined, respectively by Equations 1, 4), as well as results for both classical Hebbian and BCM learning rules. A decorrelation transition as a function of the distraction scaling parameter *s* is observed for both models and plasticity rules. In terms of the learning rules, only marginal differences are present. However, the compartment model is able to handle a significantly stronger distraction as compared to the point model. These findings support the hypothesis examined here, namely that nonlinear interactions between basal and apical input improve learning guided by top-down signals.

### 3.2. Supervised Learning in a Linear Classification Task

**Figure 2:**
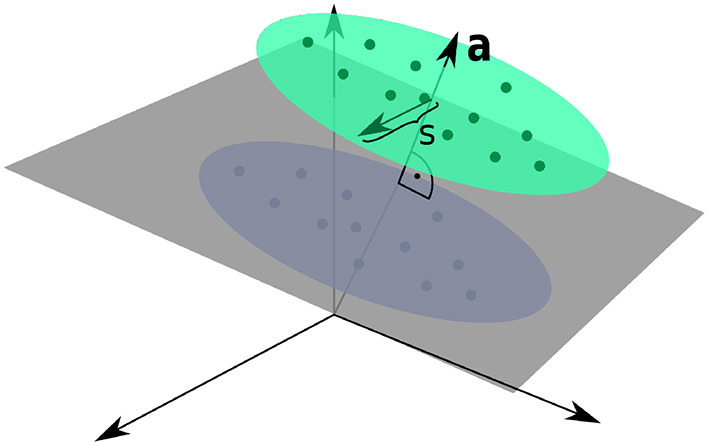
Input Space for the Linear Classification Task. Two clusters of presynaptic basal activities were generated from multivariate Gaussian distributions. Here, *s* denotes the standard deviation orthogonal to the normal vector **a** of the classification hyperplane, as defined by Equation (17).

Next, we investigated if the observed differences would also improve the performance in an actual supervised learning task. For this purpose, we constructed presynaptic basal input *x*_*p*_(*t*) as illustrated in Figure 2. Written in vector form, each sample from the basal input is generated from,

(18) $$\begin{array}{l} {\text{x}}_{p}\left(t\right)=\text{b}+\text{a}[c\left(t\right)+\sigma_{a}\zeta_{a}\left(t)\right]+s\cdot \overset{N_{\text{dist}}}{\underset{i=1}{\sum }}\zeta_{\text{dist},i}\left(t\right){\text{v}}_{\text{dist},i}, \end{array}$$

where **b** is a random vector, where each entry is drawn uniformly from [0, 1], **a** is random unit vector as introduced in section 3.1, *c*(*t*) is a binary variable drawn from {−0.5, 0.5} with equal probability and ζ_*a*_(*t*) and the ζ_dist,*i*_(*t*) are independent Gaussian random variables with zero mean and unit variance. Hence, σ_*a*_ simply denotes the standard deviation of each Gaussian cluster along the direction of the normal vector **a** and was set to σ_*a*_ = 0.25. Finally, the set of **v**_dist,*i*_ forms a randomly generated orthogonal basis of *N*_dist_ unit vectors which are—as in section 3.1—also orthogonal to **a**. The free parameter *s* parameterizes the standard deviation along this subspace orthogonal to **a**. As indicated by the time dependence, the Gaussian and binary random variables are drawn for each time step. The vectors **b**, **a**, and **v**_dist,*i*_ are generated once before the beginning of a simulation run.

For the classification task, we use two output neurons, indexed 0 and 1, receiving the same basal presynaptic input, with the respective top-down inputs *x*_*d*, 0_ and *x*_*d*, 1_ encoding the desired linear classification in a one-hot scheme,

(19) $$\begin{array}{ll} x_{d,0}\left(t\right) & =1-\Theta \left({\left({\text{x}}_{p}\left(t\right)-\text{b}\right)}^{T}\text{a}\right) \end{array}$$

(20) $$\begin{array}{ll} x_{d,1}\left(t\right) & =\Theta \left({\left({\text{x}}_{p}\left(t\right)-\text{b}\right)}^{T}\text{a}\right), \end{array}$$

where Θ(*x*) is the Heaviside step function.

As in the previous experiment, we ran a full simulation until all dynamic variables reached a stationary state. After this, a test run without plasticity and with the apical input turned off was used to evaluate the classification performance. For each sample, the index of the neuron with the highest activity was used as the predicted class. Accuracy was then calculated as the fraction of correctly classified samples.

The resulting accuracy as a function of *N*_dist_ and *s* is shown in **Figure 4**, again for all four combinations of neuron models and learning rules.

For classical Hebbian plasticity, the differences between compartmental and point neuron are small. Interestingly, the compartment model performs measurably better in the case of the BCM rule (16), in particular when the overall accuracies for the tested parameter range are compared, see **Figure 4D**. This indicates that during learning, the compartmental neuron makes better use, of the three distinct activity plateaus at 0, α and 1, when the BCM rule is at work. Compare Figure 1. We point out in this respect that the sliding threshold θ_*M*_ in (16) has been set to the point halfway between the two non-trivial activity levels, α and 1.

It should be noted that the advantage of the compartment model is also reflected in the actual correlation between proximal and distal input as a measure of successful learning (as done in the previous section), see Figure S1 in the Appendix. Interestingly, the discrepancies are more pronounced when measuring the correlation as compared to the accuracy. Moreover, it appears that above-chance accuracy is still present for parameter values where alignment is almost zero. We attribute this effect to the fact that the classification procedure predicts the class by choosing the node that has the higher activity, independent of the actual “confidence” of this prediction, i.e., how strong activities differ relative to their actual activity levels. Therefore, marginal differences can still yield the correct classification in this isolated setup, but it would be easily disrupted by finite levels of noise or additional external input.

## 4. Discussion

Pyramidal neurons in the brain possess distinct apical/basal (distant/proximal) dendritic trees. It is hence likely that models with at least two compartments are necessary for describing the functionality of pyramidal neurons. For a proposed two-compartment transfer function (Shai et al., 2015), we have introduced both unsupervised and supervised learning schemes, showing that the two-compartment neuron is significantly more robust against distracting components in the proximal input space than a corresponding (one-compartment) point neuron.

The apical and basal dendritic compartments of pyramidal neurons are located in different cortical layers Park et al. (2019), receiving top-down and feed-forward signals, respectively. The combined action of these two compartments is hence the prime candidate for the realization of backpropagation in multi-layered networks (Bengio, 2014; Lee et al., 2015; Guerguiev et al., 2017).

### 4.1. Learning Targets by Maximizing Correlation

In the past, backpropagation algorithms for pyramidal neurons concentrated on learning rules that are explicitly dependent on an error term, typically the difference between top-down and bottom-up signals. In this work, we considered an alternative approach. We postulate that the correlation between proximal and distal input constitutes a viable objective function, which is to be maximized in combination with homeostatic adaptation rules that keep proximal and distal inputs within desired working regimes. Learning correlations between distinct synaptic or compartmental inputs is as a standard task for Hebbian-type learning, which implies that the here proposed framework is based not on supervised, but on biologically viable unsupervised learning schemes.

The proximal input current *I*_*p*_ is a linear projection of the proximal input space. Maximizing the correlation between *I*_*p*_ and *I*_*d*_ (the distal current), can therefore be regarded as a form of canonical correlation analysis (CCA) (Härdle and Simar, 2007). The idea of using CCA as a possible mode of synaptic learning has previously been investigated by Haga and Fukai (2018). Interestingly, according to the authors, a BCM-learning term in the plasticity dynamics accounts for a principal component analysis in the input space, while CCA requires an additional multiplicative term between local basal and apical activity. In contrast, our results indicate that such a multiplicative term is not required to drive basal synaptic plasticity toward a maximal alignment between basal and apical input, even in the presence of distracting principal components. Apart from the advantage that this avoids the necessity of giving a biophysical interpretation of such cross-terms, it is also in line with the view that synaptic plasticity should be formulated in terms of local membrane voltage traces (Clopath et al., 2010; Weissenberger et al., 2018). According to this principle, distal compartments should therefore only implicitly affect plasticity in basal synapses, e.g., by facilitating spike initiation.

### 4.2. Generalizability of the Model to Neuroanatomical Variabillity

While some research on cortical circuits suggests the possibility of generic and scalable principles that apply to different cortical regions and their functionality (Douglas and Martin, 2007; George and Hawkins, 2009; Larkum, 2013), it is also well-known that the anatomical properties of pyramidal neurons, in particular the dendritic structure, varies significantly across cortical regions (Fuster, 1973; Funahashi et al., 1989). More specifically, going from lower to higher areas of the visual pathway, one can observe a significant increase of spines in the basal dendritic tree (Elston and Rosa, 1997; Elston, 2000), which can be associated with the fact that neurons in higher cortical areas generally encode more complex or even multi-sensory information, requiring the integration of activity from a higher number and potentially more distal neurons (Elston, 2003; Luebke, 2017).

**Figure 3:**
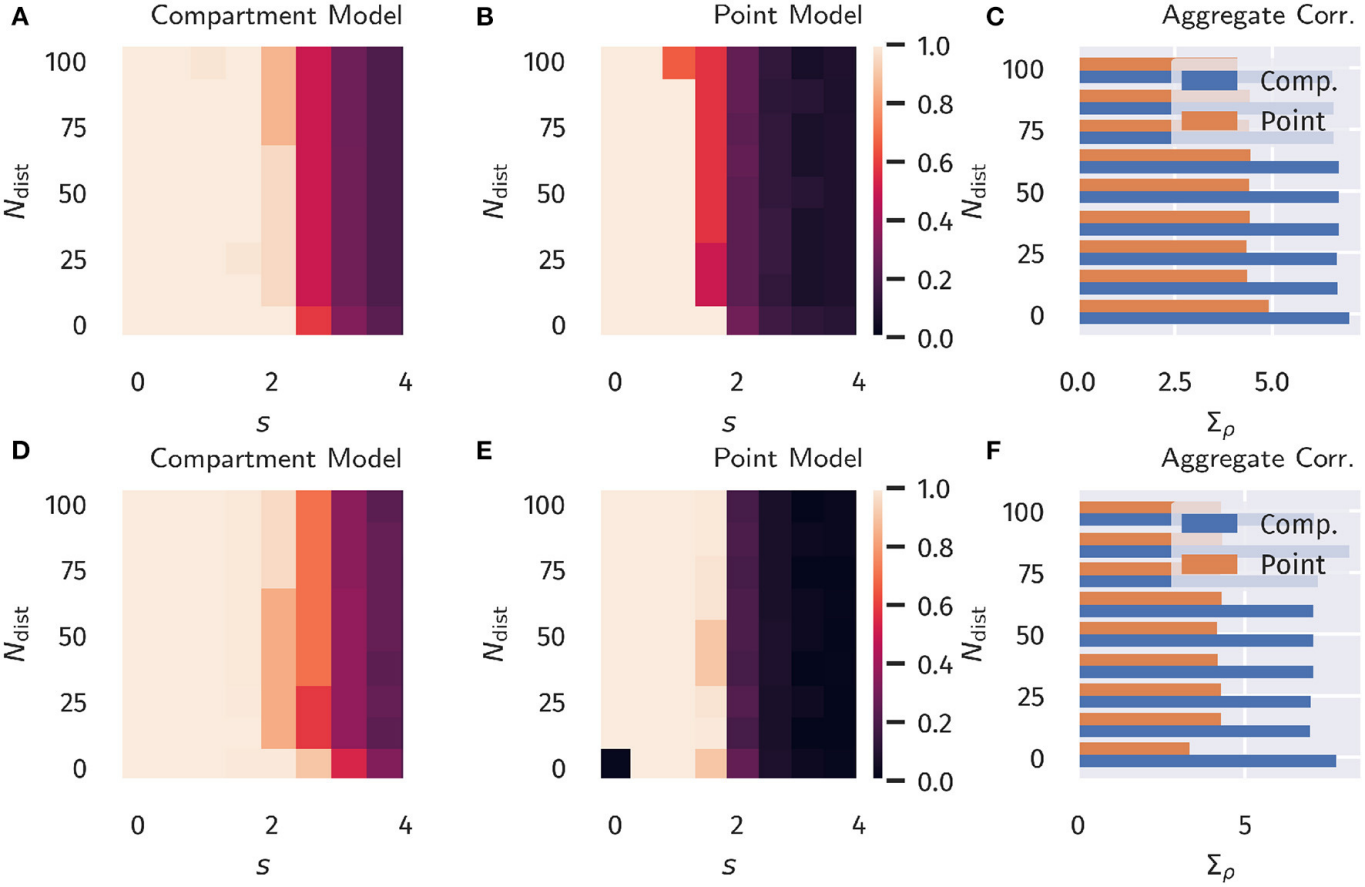
Unsupervised Alignment between Basal and Apical Input. Color encoded is the Pearson correlation ρ[*I*_*p*_, *I*_*d*_] between the proximal and distal input currents, *I*_*p*_ and *I*_*d*_. **(A–C)** Classical Hebbian plasticity, as defined by Equation (13). **(D–F)** BCM rule, see Equation (16). Data for a range *N*_dist_ ∈ [0, *N* − 1] of the orthogonal distraction directions, and scaling factors *s*, as defined in Figure 2. The overall number of basal inputs is *N* = 100. In the bar plot on the right the sum Σ_acc_ over *s* = 0, 0.5, 1.0… of the results is shown as a function of *N*_dist_. Blue bars represents the compartment model, orange the point model.

**Figure 4:**
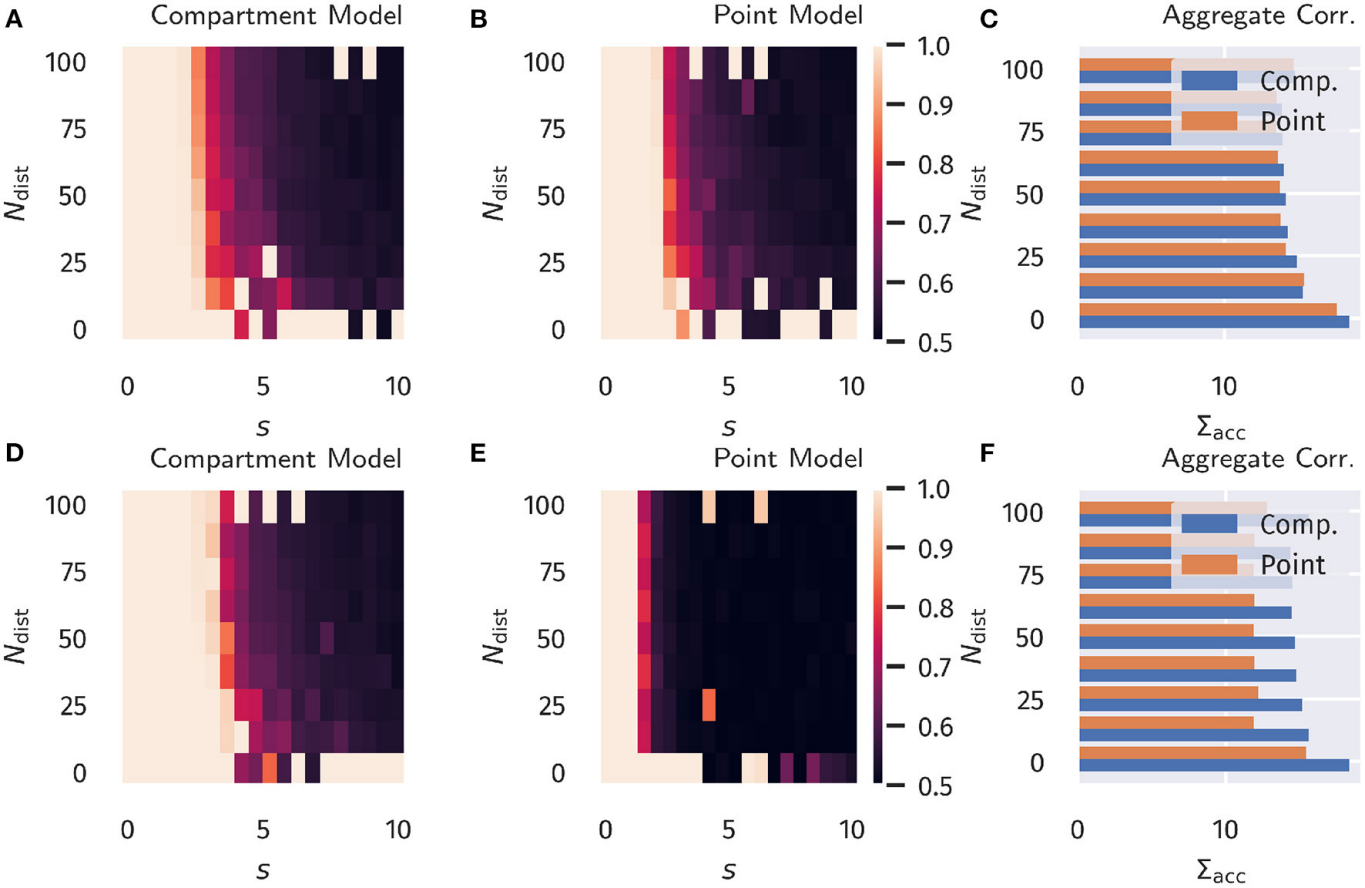
Binary Classification Accuracy. Fraction of correctly classified patterns as illustrated in Figure 2, see section 3.2. **(A–C)** Classical Hebbian plasticity. **(D–F)** BCM rule. In the bar plot on the right the sum Σ_acc_ over *s* = 0, 0.5, 1.0… of the results is given as a function of *N*_dist_. Blue bars represents the compartment model, orange the point model.

With respect to a varying amount of basal synaptic inputs, it is interesting to note that the dimensionality *N* of the basal input patterns did not have a large effect on the results of our model, see Figures 3, 4, Figure S1 in the Appendix, as long as the homeostatic processes provided weight normalization.

Apart from variations in the number of spines, variability can also be observed within the dendritic structure itself (Spruston, 2008; Ramaswamy and Markram, 2015). Such differences obviously affect the internal dynamics of the integration of synaptic inputs. Given the phenomenological nature of our neuron model, it is hard to predict how such differences would be reflected, given the diverse dynamical properties that can arise from the dendritic structure (Häusser et al., 2000). The two models tested in our study can be regarded as two extreme cases, where the point neuron represents a completely linear superposition of inputs and the compartment model being strongly nonlinear with respect to proximal and distal inputs. In principle, pyramidal structures could also exhibit properties in between, where the resulting plasticity processes would show a mixture between the classical point neuron behavior (e.g., if a dimensionality reduction of the input via PCA is the main task) and a regime dominated by proximal-distal input correlations if top-down signals should be predicted.

### 4.3. Outlook

Here we concentrated on one-dimensional distal inputs. For the case of higher-dimensional distal input patterns, as for structured multi-layered networks, it thus remains to be investigated how target signals are formed. However, as previous works have indicated, random top-down weights are generically sufficient for successful credit assignment and learning tasks (Lillicrap et al., 2016; Guerguiev et al., 2017). Therefore, we expect that our results can be also transferred to deep network structures, for which plasticity is classically guided by local errors between top-down and bottom-up signals.

## Data Availability Statement

Simulation datasets for this study can be found at: https://cloud.itp.uni-frankfurt.de/s/mSRJ6BPXjwwHmfq. The simulation and plotting code for this project can be found at: https://github.com/FabianSchubert/frontiers_dendritic_coincidence_detection.

## Author Contributions

FS and CG contributed equally to the writing and review of the manuscript. FS provided the code, ran the simulations, and prepared the figures. Both authors contributed to the article and approved the submitted version.

## Conflict of Interest

The authors declare that the research was conducted in the absence of any commercial or financial relationships that could be construed as a potential conflict of interest.

## Publisher's Note

All claims expressed in this article are solely those of the authors and do not necessarily represent those of their affiliated organizations, or those of the publisher, the editors and the reviewers. Any product that may be evaluated in this article, or claim that may be made by its manufacturer, is not guaranteed or endorsed by the publisher.
