# Supplementary material for: Nonlinear Dendritic Coincidence Detection for Supervised Learning
Source: Front Comput Neurosci. 2021 Aug 4;15:718020. doi: 10.3389/fncom.2021.718020 (PMC8372750; doi:10.3389/fncom.2021.718020)
Supplement: Supplementary file 1 [file Presentation_1.pdf]

Supplementary Material

1 ALIGNMENT IN THE CLASSIFICACTION TASK

Instead of measuring the model performance in the classification task presented in Sect. 3.2 by the fraction of correctly classified patterns, as shown in Fig. 4, one can also use the correlation between I_p and I_d, as done in Sect. 3.1. This is shown in Fig. S1. One observes a more pronounced difference between the point model and the compartment model, where the latter results in an overall better alignment for the tested parameter space.

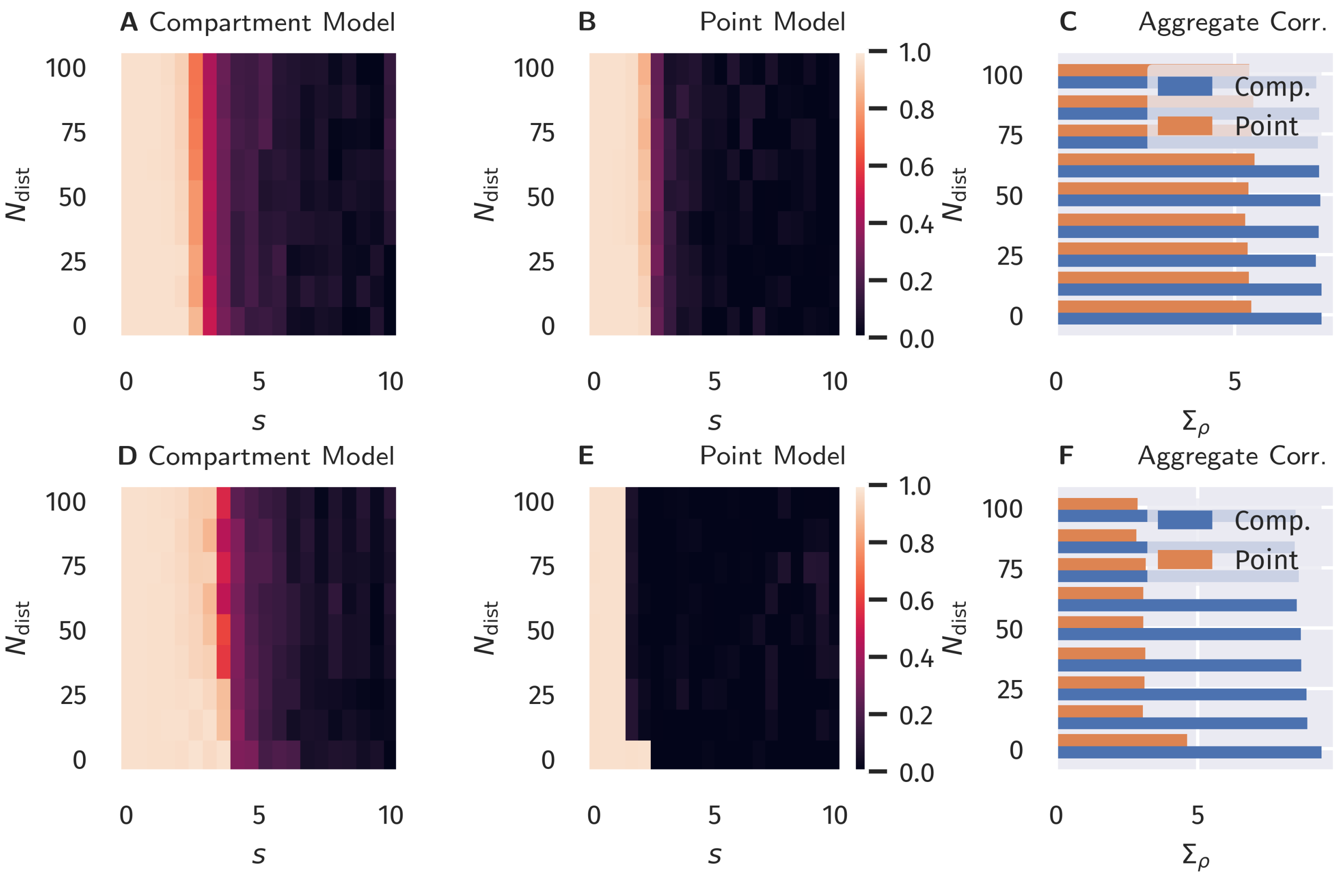

Figure S1. Alignment between Basal and Apical Input after Binary Classification Learning. Correlation between proximal and distal inputs after training, as described in Sect. 3.2. A–C: Classical Hebbian plasticity. D–F: BCM rule. In the bar plot on the right the sum $\Sigma_{\rm acc}$ over $s = 0,\, 0.5,\, 1.0$ .. of the results is shown as a function of $N_{\rm dist}$. Blue bars represents the compartment model, orange the point model.

2 OBJECTIVE FUNCTION OF BCM LEARNING IN THE COMPARTMENT MODEL

To gain a better understanding of why the BCM-type learning rule in combination with the implemented compartment model drives the neuron towards the temporal alignment between I_p and I_d, we can formalize the learning rule for the proximal weights in terms of an objective function. For this purpose, we further simplify Equation (1) by replacing the sigmoid functions $\sigma(x)$ by a simple step function $\Theta(x)$. This does not change the overall shape or topology of the activation in the (I_p, I_d) space but merely makes the smooth

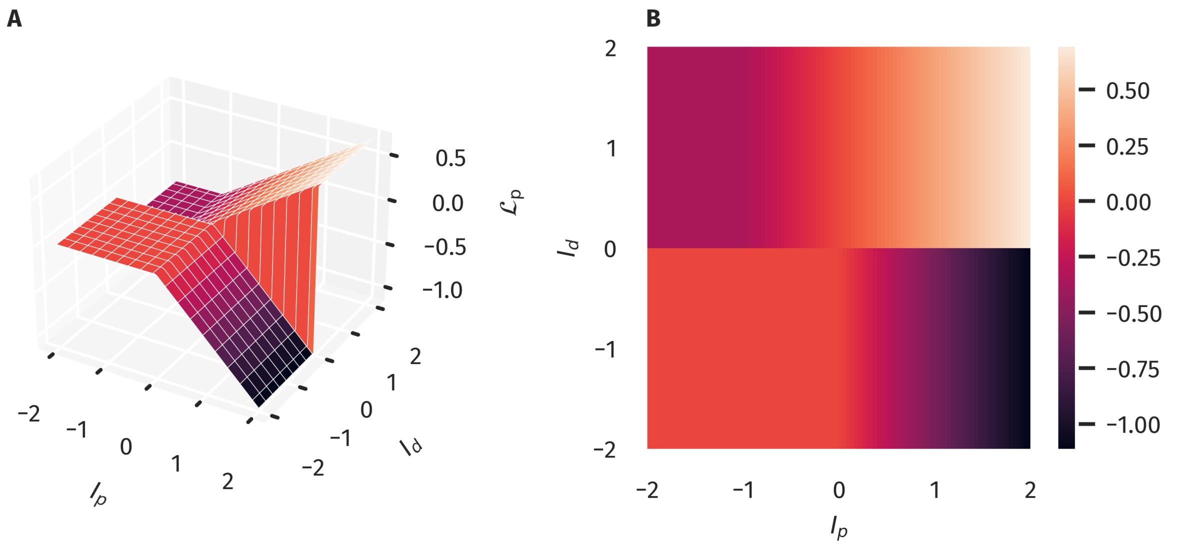

Figure S2. Objective Function for the Proximal Weight Update. The approximate objective function for the proximal weights as given in (S2) as a 3d-plot (A) and color-coded (B). This corresponds to a combination of using Equation (1) together with Equation (16). Note the ridge-like structure along the I_p-I_d diagonal, which supports the alignment between proximal and distal input.

transitions sharp and instantaneous. Using $\Delta w_i \propto y\,(y-\theta_M)\,x_i$, we find in this case

$$\Delta w_i \;\propto\; \Big[(1-\alpha)\Theta(I_d-\theta_d)\Theta(p-\theta_{p1})+\alpha(\alpha-1)\Theta(\theta_d-I_d)\Theta(p-\theta_{p0})\Big]x_i\ . \tag{S1}$$

Noting that $\Theta(x)$ is the first derivative of the ReLu function $[x]^+\equiv\max(0,x)$, we find that this update rule can be written as

$$\begin{aligned}\Delta w_i &\propto \frac{\partial \mathcal{L}_p}{\partial w_i}\\ \mathcal{L}_p &= (1-\alpha)\Theta(I_d-\theta_d)[p-\theta_{p1}]^+ + \alpha(\alpha-1)\Theta(\theta_d-I_d)[p-\theta_{p0}]^+\ .\end{aligned} \tag{S2}$$

The objective function $\mathcal{L}_p$ is shown in Fig. S2. One observes that states closer to the I_p-I_d diagonal are preferred since they tend to yield higher values of $\mathcal{L}_p$, while the opposite is the case for off-diagonal states.

It should be noted, though, that the objective function is not scale-invariant (as would be e.g. if the squared error was used) in the sense that the prior distributions of both proximal and distal inputs need a certain mean and variance to cover a region of input states for which the described effects can take place. As a counterexample, one could imagine that the input samples only covered a flat area of $\mathcal{L}_p$, as for example in Fig. S2B in the lower-left quadrant, leading to a zero average gradient. This is prevented, however, by the homeostatic processes acting simultaneously on the gains and biases, making sure that the marginal distributions of I_p and I_d are such that higher correlations are preferred. For example, if we assume a Gaussian marginal distribution for both I_p and I_d with zero means and a standard deviation of 0.5 (which is used as a homeostatic target in the simulations), the expected value of $\mathcal{L}(I_p, I_d)$ is -0.055 if I_p and I_d are completely uncorrelated, and 0.07 in the perfectly correlated case.
